# 

**Published:** 1874-11

**Authors:** 


### CONTENTS:

### ORIGINAL COMMUNICATIONS.

ARTICLE I.—Annual Address before the American Academy of Dental Science. By Dr. W. W. Allport ... 289

ARTICLE II.—Report of the Committee on Mechanical Dentistry, To Southern Dental Association, 1874 ... 301

### SELECTED ARTICLES.

ARTICLE III.—Proceedings of American Dental Association ... 305

ARTICLE IV.—The Rubber Dam ... 318

ARTICLE V.—Excision of the Superior Maxillary. By D. Keller, M. D ... 324

### EDITORIAL, ETC.

The Thirty-Fifth Annual Session of the Baltimore College of Dental Surgery ... 325

Spongoid ... 326

Salivary Calculus. Defective Palate, Effects of Inferior Teeth upon Alveolar Process of Superior Maxillary and Exostosed Teeth ... 329

### BIBLIOGRAPHICAL.

Chemical Lectures on Diseases of Nervous System ... 330

Instructions in Manipulation of Hard Rnbber or Vulcanite ... 330

The Physicians Visiting List for 1875 ... 330

### MONTHLY SUMMARY.

The Connection of Cancer and Skin Disease ... 331

Gelseminum in Odontalgia ... 332

How to "Pull" Teeth ... 332

Oleate of Mercury in Treatment of Ringworm ... 333

A Lost Case ... 333

To Suppress Hemorrhage from the Mouth during Operations ... 334

The Medical Education of Women ... 334

Longevity of Medical Men ... 334

Cremation ... 334

Table-fork Swallowed and Discovered in Stomach 5 or 6 Years Afterwards ... 335

The Danger of Mixing Chromic Acid with Glycerine ... 335

